# High-Sensitivity Electrical Admittance Sensor with Regression Analysis for Measuring Mixed Electrolyte Concentrations

**DOI:** 10.3390/s24227379

**Published:** 2024-11-19

**Authors:** Chun-Chi Chen, Chih-Hung Hung, Han-Xiang Zhu, Ji-Zun Chen

**Affiliations:** Electrical Engineering Department, National Chiayi University, Chiayi 600355, Taiwan

**Keywords:** electrolyte concentration measurement, point-of-care diagnosis, healthcare monitoring system

## Abstract

Electrolyte balance is essential for the proper functioning of the body, and imbalances can lead to various health issues, some of which may be life-threatening. Therefore, measuring electrolyte concentrations is becoming increasingly important, particularly for athletes engaged in high-intensity and prolonged physical activity. In this project, we developed a highly sensitive sensing device capable of accurately and rapidly measuring electrolyte concentrations in mixed solutions, providing precise analysis of trace electrolyte levels. The sensor device requires no complex operational procedures and can quickly complete measurements, making it well-suited for point-of-care applications. Integration of regression models further enhances the device’s ability to estimate concentrations in mixed electrolyte solutions. The test results demonstrate that the device can detect subtle concentration variations, with a precision as low as 0.5 mM. This proposed sensing device offers a cost-effective and efficient solution for real-time monitoring of electrolyte levels in healthcare.

## 1. Introduction

In biomedical research and healthcare, electrolyte measurement is crucial for the early detection, diagnosis, and management of various health conditions. Electrolyte imbalance in the body can lead to a wide range of health problems and symptoms. Specifically, imbalances in potassium, sodium, or magnesium levels can result in conditions such as hypernatremia, cardiac arrhythmias, and dehydration [1,2,3]. Moreover, athletes engaged in intense physical activities who lose fluids and electrolytes through sweat may face serious health risks. These include kidney injury, brain damage, and cardiovascular strain [4,5,6]. In extreme cases, electrolyte imbalances can be life-threatening, potentially leading to seizures, coma, or cardiac arrest [7,8]. In the field of point-of-care diagnostics and healthcare monitoring systems, electrolyte measurement aids in monitoring patient health by providing early identification of electrolyte imbalances. Human body fluids, such as sweat, saliva, tears, and blood, are rich sources of health parameters crucial for assessing the body’s overall status. For instance, monitoring electrolyte concentrations in body fluids provides crucial insights into various medical conditions, including electrolyte imbalances, kidney disorders, and cardiovascular diseases [9,10,11]. However, obtaining biomarkers through blood collection is an invasive procedure that can cause discomfort, pain, or anxiety for some individuals, and the act of needle insertion carries the risk of complications such as bruising and infection. Moreover, blood tests require specific medical supplies and the involvement of skilled healthcare professionals, resulting in significant costs and resource demands. As the demand for healthcare monitoring systems continues to grow, noninvasive electrolyte measurements become increasingly critical for personalized healthcare and treatment strategies.

Traditional methods of electrolyte measurement, such as Inductively Coupled Plasma–Mass Spectrometry (ICP-MS), Near Infrared Spectroscopy (NIR), Ion Chromatography, and Flame Photometry, have limitations, including high setup and analysis costs, extensive and complex sample preparation requiring large volumes, along with intricate operating procedures [12,13,14,15,16]. Electrochemical Impedance Spectroscopy (EIS) measures electrolyte concentration by analyzing the impedance of an electrochemical cell across a range of AC frequencies, providing sensitive and detailed analysis [17,18]. However, the electrodes often involve redox (reduction–oxidation) reactions, which may impact long-term stability and limit applications requiring a non-destructive approach. Additionally, EIS can be time-consuming due to the need for broad-frequency data collection and requires costly equipment that demands regular calibration and maintenance, limiting its accessibility for field-deployable applications. While Ion-Selective Electrodes (ISEs) offer a more cost-effective electrochemical approach, they are constrained to measuring specific ions and often suffer from low sensitivity. Moreover, the lifespan of the electrode and selective membrane may degrade over time, impacting the reliability and accuracy of measurements along with requiring regular calibration [19,20,21]. Although recent developments have miniaturized ISE sensors for wearable devices, the fabrication process for these miniature ISEs is complex and costly [22,23,24]. The electrodes require pre-treatment with a conductive inner layer, and the use of precious metals and specialized materials further raises production costs. Each electrode membrane is generally limited to detecting a specific ion, and achieving ion equilibrium can prolong testing times. Furthermore, finding suitable ion-selective membranes for certain target electrolytes remains challenging.

In this work, we developed an electrical sensor device that is highly sensitive to electrolyte concentration, allowing for accurate measurements even at very low levels. This compact and portable sensor device enables rapid and precise measurement of electrolyte concentrations in mixed solutions. Our sensor employs an admittance-based approach combined with real-time regression analysis, offering advantages in sensitivity, simplicity, and rapid measurement. Unlike traditional EIS systems that require scanning across a broad frequency range, our sensor operates efficiently at specific frequencies, simplifying the measurement process. To address potential inaccuracies in traditional sensors caused by load impedance variations [25], we implemented a comparator-based operational amplifier to enhance the detection and measurement of subtle admittance changes with high precision. In addition, an insulating layer between the electrode and sample prevents electrochemical reactions, ensuring non-destructive testing. Our design requires only a small sample volume and allows measurements in mixed solutions without restrictions to specific ions and without restrictions to specific electrolyte solutions. Its compact, low-cost construction makes it suitable for mass production and field deployment. Its compact and low-cost design makes it suitable for mass production and field deployment. The test results demonstrate its high sensitivity and accuracy, making it suitable for field-deployable measurements in healthcare applications.

## 2. Materials and Methods

To measure electrolyte concentrations, we have designed a sensor device capable of rapidly detecting electrical responses corresponding to the electrolyte levels. This compact and portable sensor facilitates its application across various settings, including point-of-care and field-based measurements. It can be utilized for health status assessments, particularly in clinical settings where electrolyte imbalances can critically affect patient outcomes. To enhance the device’s sensitivity, we have integrated a comparator-based amplifier into the system. This integration ensures that even the most subtle changes in electrolyte concentrations are accurately detected and amplified, allowing for precise and reliable measurements. Further details regarding the design and functionality of our proposed rapid-response electrolyte sensor are provided in the following subsections.

### 2.1. Admittance Sensing Device

**Figure 1:**
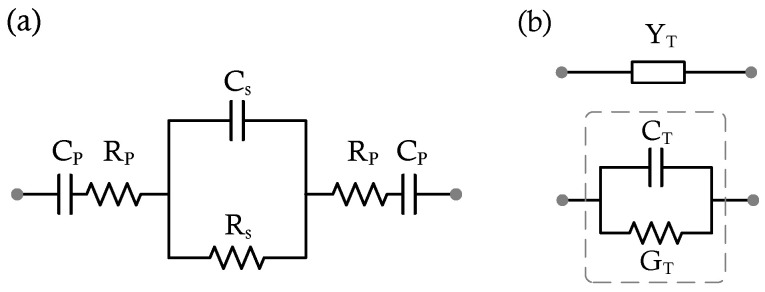
Equivalent circuit model for an electrolyte sample. (**a**) The equivalent circuit consists of the polarization impedance of the electrodes, represented by $R_{P}$ and $C_{P}$, along with the sample impedance, represented by $R_{S}$ and $C_{S}$. (**b**) The simplified equivalent circuit consists of the equivalent admittance $Y_{T}$, represented by conductance $G_{T}$ and capacitance $C_{T}$.

An electrolyte sample consists of a solvent, typically water, in which electrolytes are dissolved to form free ions. These ions, comprising both cations and anions, facilitate the transfer of electrical charge through the solution, providing electrolyte solutions with high conductivity. Moreover, when a voltage is applied across the electrodes, these ions migrate toward the electrode interface under the influence of an electric field. This ion migration leads to the accumulation of ions at the electrode surfaces, inducing electrode polarization and forming an electrical double layer [26]. The distribution and behavior of ions within the electrical double layer is influenced by the applied voltage and ion concentration. Additionally, different electrolyte solutions exhibit unique polarization impedance due to variations in ion mobility, charge density, and specific electrochemical interactions at the electrode interface. The distinct impedance behavior for each electrolyte enables differentiation among electrolyte types when a voltage is applied. Furthermore, the electrolyte sample subjected to voltage, forming an electrical double layer, can be modeled as an equivalent circuit [27], as illustrated in Figure 1a. In this model, the polarization impedance of the electrodes can be expressed as $Z_{P}=R_{P}+\frac{1}{jwC_{P}}$, where *j* is the imaginary unit and *w* represents angular frequency. The symmetric polarization impedances correspond to identical electrodes, and these impedances are arranged in series with the sample impedance denoted as $Z_{S}=R_{S}\;\|\;C_{S}$. The series resistance and capacitance model can be further simplified to an admittance circuit model, as shown in Figure 1b, assuming the electrode polarization is balanced and stable. Based on this model, analyzing the admittance response at a fixed voltage allows for the accurate detection and quantification of variations in electrolyte concentration. This technique not only simplifies the measurement process but also enhances the reliability of electrolyte concentration assessments.

**Figure 2:**
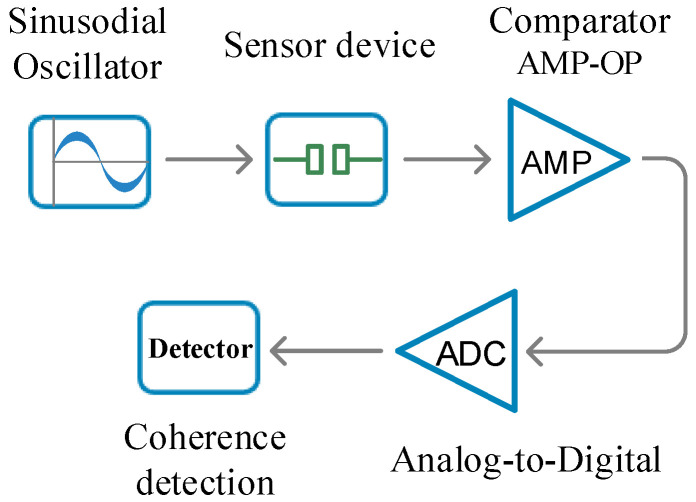
The electrolyte sensing system. The sinusoidal oscillator generates a sinusoidal signal to test the samples on the sensor device. The testing signal response is then amplified by a comparator-based operational amplifier (op-amp) and converted into digital signals by an analog-to-digital converter (ADC) for precise and coherent detection.

**Figure 3:**
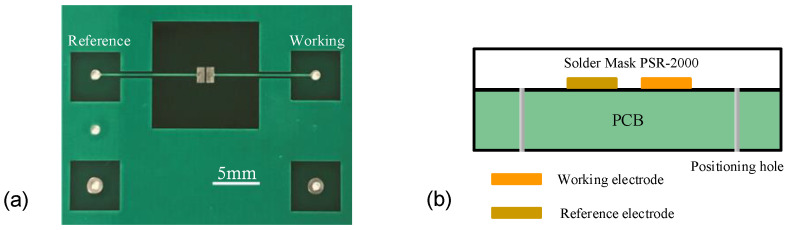
The electrode sensing device. (**a**) The sensor device comprises coplanar copper electrodes on the PCB, utilizing a working electrode and a reference electrode array for measurements. The PCB also incorporates corner holes to ensure precise positioning. (**b**) The cross-sectional diagram of the sensing device shows the sensing electrodes embedded within the copper foil layer, covered with a thin insulation layer (PSR-2000) on the PCB.

The proposed sensing system is demonstrated in Figure 2. To analyze the sample on the sensor device, the system utilizes an oscillator to generate a sinusoidal signal at a predetermined working frequency. The admittance of the test sample varies with electrolyte concentration, resulting in different impedance responses that alter the output signal accordingly. The signal response is amplified using a comparator-based operational amplifier (op-amp) to capture the minute variations in the sample. Subsequently, this amplified signal is converted into a digital format for coherent amplitude detection, enabling precise evaluation of electrolyte concentration in the test sample [28,29]. The proposed sensing electrode device, designed with coplanar electrodes for measuring electrolyte concentration, is illustrated in Figure 3. The sensing electrode device is integrated onto a Printed Circuit Board (PCB), primarily composed of fiberglass and resin matrix. This sensing device utilizes coplanar electrodes, including a working electrode and a reference electrode array, for electrolyte measurement. Coplanar electrode sensors exhibit high sensitivity to changes in the electrical properties of samples, making them suitable for detecting minute changes in concentration or the presence of specific compounds [30,31]. The sensing electrodes are embedded within the PCB’s copper foil layer, with widths of 900 μm, lengths of 1800 μm, and a gap of 200 μm between them. To prevent chemical interactions and redox reactions during testing, a thin insulation layer of PSR-2000, with a depth of 10 μm, was applied over the copper foil layer as illustrated in Figure 3b. This layer isolates the test sample from direct contact with the sensing electrode, thereby eliminating potential biases caused by physical interactions and improving the accuracy of electrolyte measurements. The conventional three-electrode system, which includes a working electrode, reference electrode, and counter electrode, allows for the control of reactions in solution. Molecules in the solution undergo redox reactions at the working electrode, generating a measurable current, while the reference electrode provides a stable potential baseline. Meanwhile, the counter electrode balances the current generated by the working electrode, maintaining consistent current flow within the system to support stable electrochemical reactions. In contrast, our configuration incorporates an insulating layer and lacks direct conductive electrodes to prevent electrochemical reactions. Since no electrochemical reaction occurs, this design enables stable admittance measurements without requiring traditional electrode arrangements. Furthermore, the sensing device is manufactured using standard PCB fabrication techniques without specialized materials, making it cost-effective and suitable for mass production.

### 2.2. Comparator-Based Amplifier

**Figure 4:**
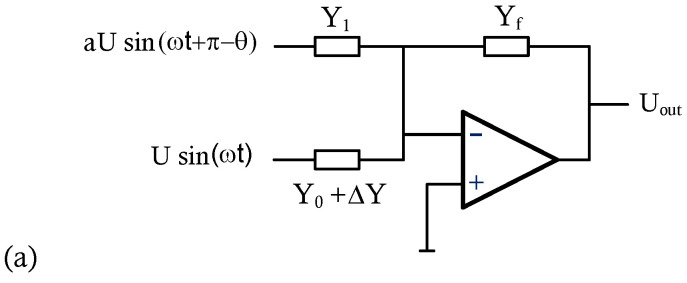

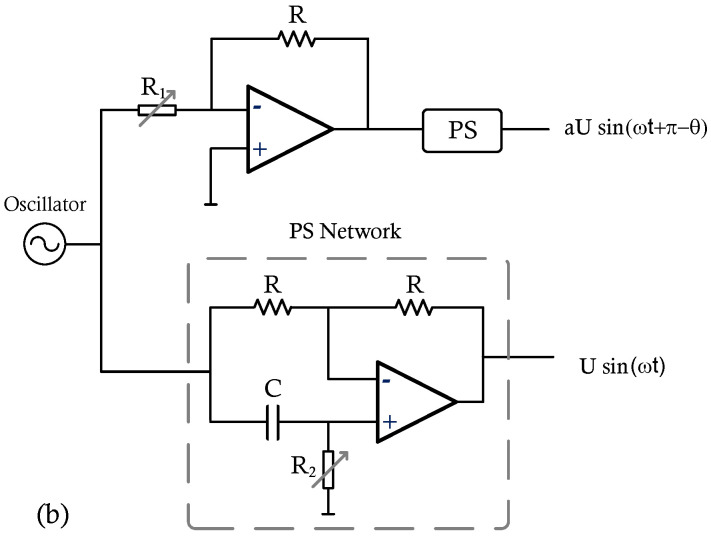
The comparator−based operational amplifier. (**a**) The comparator-based op-amp circuit amplifies the difference in signal response between the target admittance $Y_{0}+\Delta Y$ and the reference admittance $Y_{1}$. (**b**) The circuit of the signal generator produces two-phase driving signals with a 180° phase difference. The inverting op-amp can control the output amplitude by adjusting the variable resistor $R_{1}$. The Phase Shift (PS) networks can adjust the phase to synchronize the signal and minimize the phase difference by utilizing the variable resistance $R_{2}$.

The comparator-based op-amp utilizing negative feedback offers improved stability and linearity in the output signal, leading to more accurate signal amplification [32,33]. Characterized by its high-gain and low-noise amplification, it is suitable for a variety of applications, including function generators, audio mixers, capacitance-to-phase conversion, and Analog-to-Digital Converter (ADC) [34,35,36]. To increase the sensitivity of the measurement, we incorporated a comparator-based amplifier into our sensing device, as depicted in Figure 4. This approach effectively reduces the impact of common admittance and environmental biases, thereby achieving higher linearity and an extended dynamic range for amplification. Furthermore, the op-amp with negative feedback can enhance closed-loop input and output impedances, ensuring the accuracy and reliability of target admittance measurements. Therefore, this design can detect small variations in admittance between reference and target samples, enabling precise measurement and analysis of electrolyte concentrations. The driving circuit, illustrated in Figure 4b, generates the two-phase driving signals with a 180-degree phase difference. It employs an inverting op-amp to produce the inverted phase signal, utilizing the variable resistor $R_{1}$ to control the amplitude of the driving signal. The Phase Shift (PS) networks within the circuit are designed to adjust and synchronize the phases, effectively minimizing phase differences between the driving signals. The voltage gain of the PS network can be expressed in terms of phasors:

(1) $$\frac{V_{o}}{V_{i}}=\frac{j\omega R_{2}C-1}{j\omega R_{2}C+1},$$

where $V_{i}$ and $V_{o}$ are the input and output sinusoidal signals with a fixed frequency, *w* is the corresponding angular frequency, and $R_{2}$ is a variable resistor used to adjust the phase difference. By adjusting the variable resistors within the PS networks, the phase shift between the driving signals can be finely balanced to minimize the mismatch. Consequently, the two-phase driving signals Usin(ωt) and aUsin(ωt+π−θ) are generated to test the target samples, where *U* and aU are the amplitudes of the signals. Assuming the phase mismatch θ is small, the synchronized two-phase driving signals can be represented in terms of phasors as *U* and $-aU(1-\theta^{2}/2-j\theta ))$. With the admittance $Y_{0}=aY_{1}$ and feedback admittance $Y_{f}$, the output response of the comparator-based amplifier can be expressed as

(2) $$\begin{array}{cc} U_{out} & =-\left[U\left(Y_{0}+\Delta Y\right)-aUY_{1}\left(1-\theta^{2}/2-j\theta \right)\right]/Y_{f}\\ \; & =-\left[\Delta Y+Y_{0}\left(\theta^{2}/2+j\theta \right)\right]U/Y_{f}. \end{array}$$

Consider the admittance change ΔY=ΔG+jΔB, where ΔG and ΔB represent the changes in conductance and susceptance, respectively. Equation (2) can then be rearranged as follows:

(3) $$\begin{array}{cc} U_{out} & =-\left[\Delta G+j\Delta B+Y_{0}\left(\theta^{2}/2+j\theta \right)\right]U/Y_{f}\\ \; & =-\left[\Delta G+Y_{0}\theta^{2}/2+j\left(\Delta B+Y_{0}\theta \right)\right]U/Y_{f}. \end{array}$$

Based on the in-phase and quadrature components outlined in Equation (3), the changes in conductance and susceptance can be amplified by a factor of $U/Y_{f}$. Although fixed biases $Y_{0}\theta^{2}/2$ and $Y_{0}\theta$ are present, they can be eliminated by adopting the initial measurement $Y_{0}$ as the reference. To achieve a large amplifying factor for the measurement, the driving voltage *U* should be large, and the feedback admittance $Y_{f}$ should be small. However, the maximum driving voltage is constrained by the oscillator, and the feedback admittance must be limited to prevent overloading. By properly adjusting the amplifying factors to control the frequency response, the comparator-based amplifier can detect minuscule changes in the admittance of the test samples at different frequencies. This allows for high sensitivity and accuracy in measuring the corresponding electrolyte concentrations.

## 3. Results and Discussion

The sensor device prototype was assembled using standard OP37 op-amps (Analog Devices, Norwood, MA, USA) and a PicoScope-2208B USB oscilloscope (Pico Technology, St Neots, Cambridgeshire, UK), which served as the ADC to facilitate coherent detection and further experimental analysis. Given that the sampling rate of 1 MHz is common for ADCs used in various applications, it could be substituted with a dedicated ADC chip and microcontroller customized for specific applications. The admittance response was analyzed using computer-based programmable software for electrolyte concentration estimation. For the driving signals, the reference voltage U=500 mV was applied, and the two-phase driving signals were adjusted to satisfy the admittance condition $Y_{0}=aY_{1}$ for the target samples. For enhanced amplification, the feedback admittance was configured with a resistance of 200 kΩ in parallel with a capacitance of 100 pF. The capacitance allows for different frequency responses, enabling the system to achieve measurement diversity in detecting subtle changes in admittance across a range of frequencies.

### 3.1. Electrolyte Response

As sweat is a potential source of next-generation biomarkers containing primary electrolytes such as sodium, potassium, and chloride [37,38], we focused on testing our proposed sensing device using sodium chloride (NaCl) and potassium chloride (KCl) solutions in the experiments. Extra pure NaCl with 99.9% purity and KCl with 98% purity were diluted in deionized water to assess the admittance changes in relation to the concentration of the electrolyte solutions. For this, five samples of each solution were prepared, each with a volume of 20 μL and concentration levels ranging from 0.5 mM to 4 mM. The admittance changes for these samples were then measured by the sensor device. Additionally, deionized water served as the reference solution to measure the admittance changes due to the slight variations in concentration. The measurements were conducted within a temperature range of 26–27 °C and a relative humidity between 45 and 55%.

**Figure 5:**
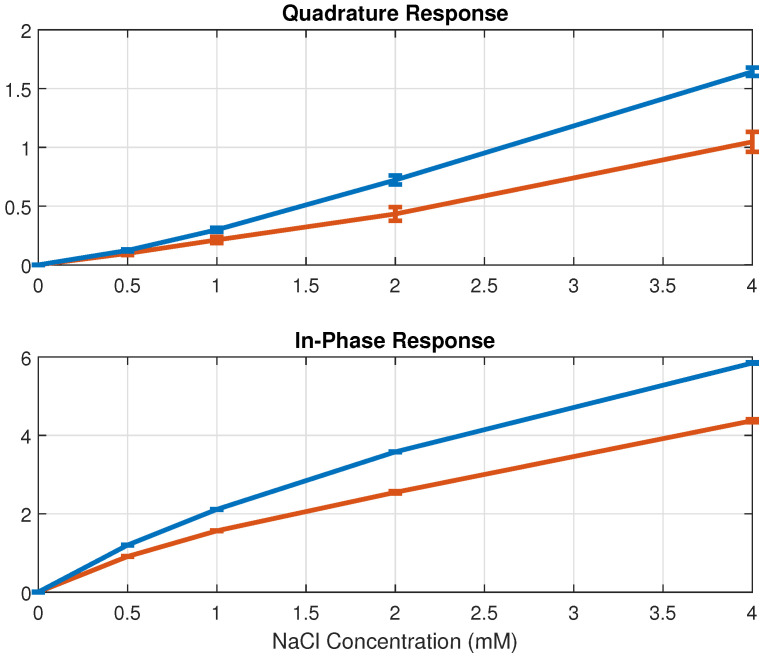
The admittance responses for deionized water and diluted NaCl solutions across concentrations ranging from 0.5 to 4 mM are presented, with the blue line measured at 5 kHz and the red line measured at 10 kHz. The responses are normalized using deionized water as the reference, and each error bar shows the standard deviation of the measurements.

**Figure 6:**
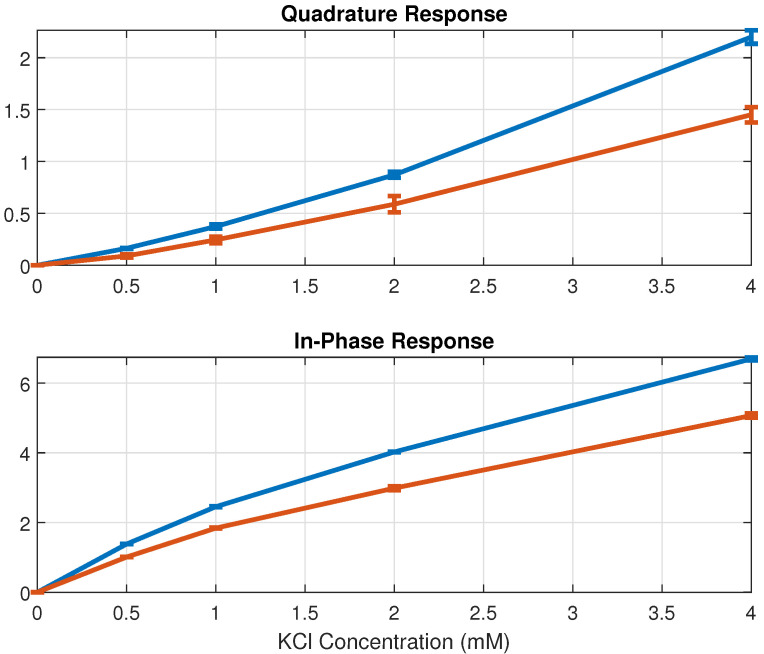
The admittance responses for deionized water and diluted KCl solutions across concentrations ranging from 0.5 to 4 mM are presented, with the blue line measured at 5 kHz and the red line measured at 10 kHz. The responses are normalized using deionized water as the reference, and each error bar shows the standard deviation of the measurements.

Figure 5 and Figure 6 illustrate the admittance response of the in-phase and quadrature components for deionized water, along with diluted NaCl and KCl solutions at various concentrations, measured at 5 kHz and 10 kHz, respectively. As the in-phase response reflects conductance change ΔG and the quadrature response reflects capacitance change ΔC, increases in admittance are observed as the electrolyte concentrations rise. Furthermore, the capacitance change exhibits an approximately linear relationship with electrolyte concentration. Therefore, variations in electrolyte concentration can be estimated from the sensing capacitance response using linear models. The correlation coefficients for NaCl are R=0.997 at 5 kHz and R=0.996 at 10 kHz, while for KCl, they are R=0.993 at 5 kHz and R=0.994 at 10 kHz (significant p<0.001). As demonstrated in Figure 5 and Figure 6, the sensor device effectively measures admittance changes at low electrolyte concentrations through both in-phase and quadrature responses, indicating its sensitivity to subtle changes in electrolyte concentration.

Due to the smaller hydration radius compared to sodium ions, potassium ions have higher mobility, resulting in greater migration efficiency in an electric field. Consequently, potassium ion solutions can exhibit slightly higher conductivity than sodium ion solutions at the same concentration, although this difference is subtle and requires precise measurement for accurate observation. In addition, the smaller hydration radius of potassium ions allows them to approach the electrode surface more closely. This reduces the distance between ions and the electrode, resulting in a denser charge distribution at the interface and an enhanced capacitance response. By utilizing the unique impedance responses of each electrolyte type, we can effectively differentiate among various electrolyte solutions.

### 3.2. Mixed Electrolyte Response

**Figure 7:**
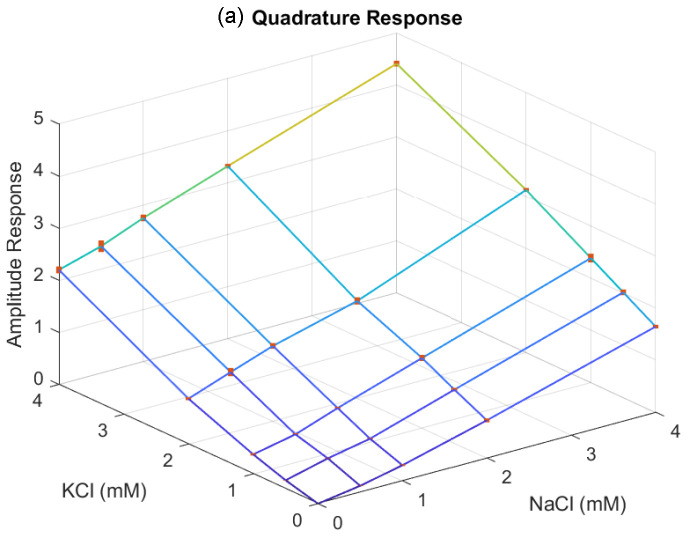

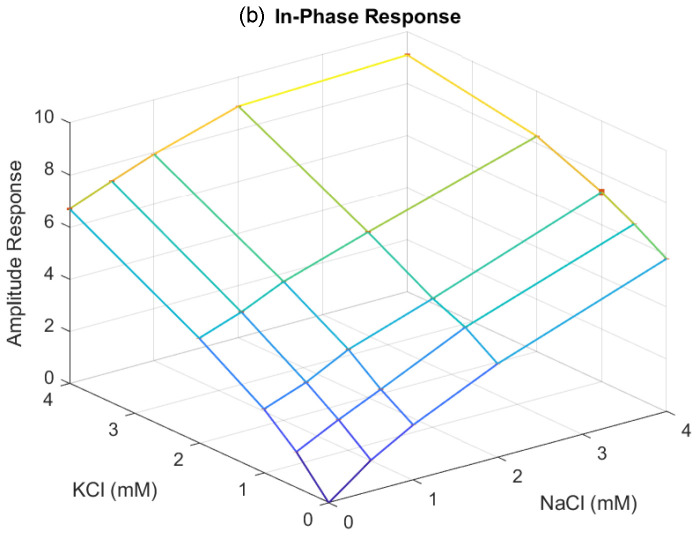
The admittance measured at 5 kHz responses for deionized water and mixed NaCl and KCl solutions across concentrations ranging from 0.5 to 4 mM. The (**a**) quadrature response and (**b**) in-phase response are normalized using deionized water as the reference solution. Each error bar indicates the standard deviation of the measurements.

**Figure 8:**
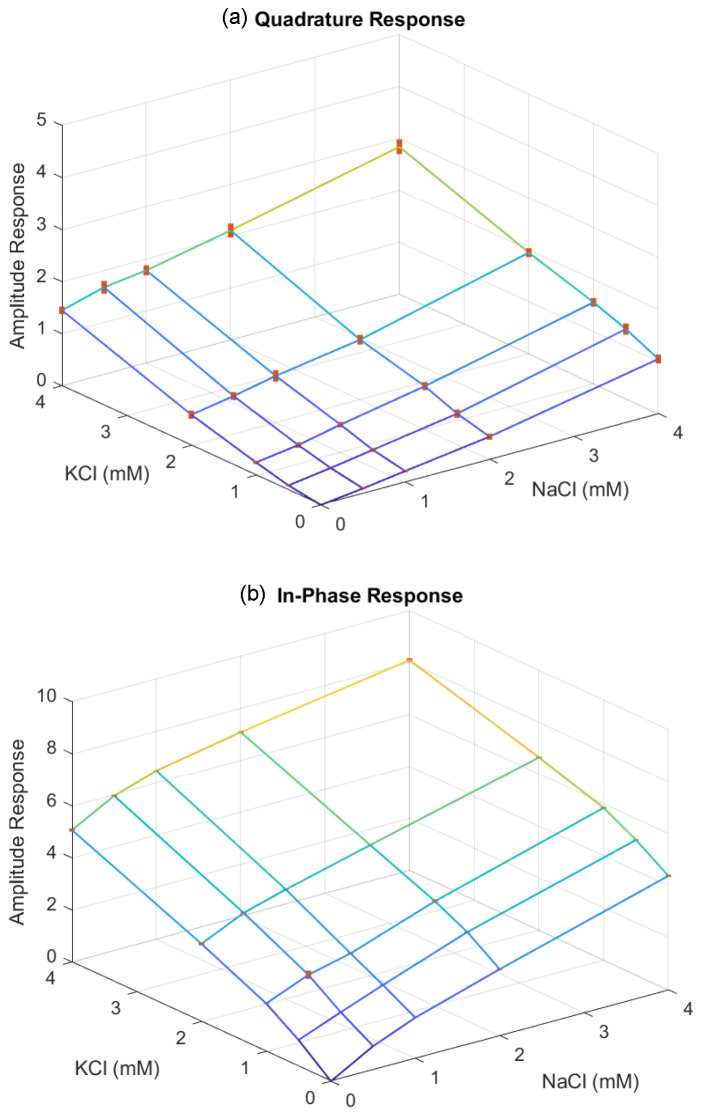
The admittance measured at 10 kHz responses for deionized water and mixed NaCl and KCl solutions across concentrations ranging from 0.5 to 4 mM. The (**a**) quadrature response and (**b**) in-phase response are normalized using deionized water as the reference solution. Each error bar indicates the standard deviation of the measurements.

To investigate the admittance responses in relation to the mixed electrolyte concentrations, we further tested solutions combining NaCl and KCl, with concentrations ranging from 0.5 mM to 4 mM. Figure 7 and Figure 8 illustrate the admittance responses for the in-phase and quadrature components of deionized water and the mixed solutions at different concentrations, measured at frequencies of 5 kHz and 10 kHz. The admittance responses demonstrate consistent increases in both conductance and capacitance as the mixed electrolyte concentrations increase. Based on the quadratic regression analysis, the in-phase and quadrature responses can be modeled using the quadratic equation

(4) $$\begin{array}{cc} V_{o} & =\alpha_{0}+\alpha_{1}x_{1}+\alpha_{2}x_{1}^{2}+\alpha_{3}x_{2}+\alpha_{4}x_{2}^{2}, \end{array}$$

where $x_{1}$ and $x_{2}$ are the concentrations of NaCl and KCl, respectively, in millimolar. The quadratic model fitting coefficients for the in-phase and quadrature responses are presented in Table 1. Using the quadratic model, the mixed electrolyte concentrations can be estimated through the Newton–Raphson approach. Additionally, the capacitance change exhibits a similar linear relationship with the mixed electrolyte concentration, further facilitating concentration estimation. The linear fitting coefficients for the in-phase and quadrature responses are presented in Table 2, with the quadrature responses showing a better fit to the linear model. Hence, the linear model of the quadrature response can be combined with the quadratic model of the in-phase response to estimate mixed concentrations. Although it is possible to use a single frequency for mixed concentration measurement, certain concentration combinations may result in non-unique solutions, leading to failure in concentration estimation. With two-frequency measurements, linear models for quadrature responses can directly estimate the mixed concentrations. Furthermore, these linear solutions can serve as initial points for quadratic models in the Newton–Raphson approach, refining the estimation and improving accuracy. This approach mitigates the issue of non-unique solutions commonly encountered with single-frequency measurements, providing a more reliable and robust method for concentration estimation.

## 4. Conclusions

In this paper, we developed a rapid, cost-effective electrical sensing device for measuring electrolyte concentrations. By utilizing coplanar electrodes for sensing admittance and a comparator-based operational amplifier for signal amplification, the sensor can detect subtle changes in admittance associated with variations in electrolyte levels. The test results show that the sensor can accurately detect small changes in capacitance and conductance corresponding to concentration variations, demonstrating its high sensitivity. The integration of regression models further improves its capability to estimate concentrations in mixed electrolyte solutions. Although the measurement requires establishing regression models in advance, the pre-built analysis model can simplify the process, making the final estimation easier to implement. Consequently, the sensor device is highly sensitive to mixed electrolyte measurement, and its cost-effective design makes it suitable for widespread healthcare screening. The proposed device offers a rapid, economical solution for monitoring electrolyte levels, with potential improvements in healthcare management and diagnostic practices.

## 5. Future Work

In future work, we aim to enhance this sensor device by integrating an ADC and microcontroller for real-time data processing, enabling autonomous and on-site electrolyte concentration analysis. A broader range of electrolyte solutions will be tested at specific frequencies to identify corresponding impedance responses. Additionally, the measurement frequency range of the sensor device will be expanded to allow for detailed impedance spectra to analyze complex solutions, including mixed-valence ion solutions. These advancements will make the sensor more valuable for point-of-care and continuous monitoring applications.

## Figures and Tables

**Table 1:** Quadratic model regression analysis.

Freq.	Response	Coefficeints	*R*
5 k	Quadrature	[−0.169, 0.438, 0.016, 0.519, 0.025]	0.998
5 k	In-phase	[0.684, 1.604, −0.120, 1.816, −0.117]	0.985
10 k	Quadrature	[−0.08, 0.236, 0.020, 0.309, 0.023]	0.998
10 k	In-phase	[0.367, 1.275, −0.083, 1.503, −0.089]	0.995

**Table 2:** Linear model regression analysis.

Freq.	Response	Coefficeints	*R*
5 k	Quadrature	[−0.246, 0.502, 0, 0.62, 0]	0.997
5 k	In-phase	[1.143, 1.110, 0, 1.333, 0]	0.979
10 k	Quadrature	[−0.163, 0.318, 0, 0.405, 0]	0.996
10 k	In-phase	[0.7, 0.932, 0, 1.137, 0]	0.990

## Data Availability

The regression data used in this study are available at https://github.com/aky3100/Sensor4Electrolyte (accessed on 15 November 2024).

## Abbreviations

The following abbreviations are used in this manuscript:

ICP-MS	Inductively coupled plasma–mass spectrometry
NIR	Near-infrared spectroscopy
ISE	Ion-selective electrode
OP-AMP	Operational amplifier
PCB	Printed circuit board
ADC	Analog-to-digital converter
PS	Phase shift
NaCl	Sodium chloride
KCl	Potassium chloride
